# Results of a cognitive behavior therapy-based intervention for antenatal anxiety on birth outcomes in Pakistan: a randomized control trial

**DOI:** 10.1038/s41598-024-64119-z

**Published:** 2024-06-14

**Authors:** Kirsten F. Siebach, Jamie Perin, Abid Malik, Najia Atif, Ahmed Zaidi, Atif Rahman, Pamela J. Surkan

**Affiliations:** 1 Department of International Health, Johns Hopkins School of Public Health, 615 N. Wolfe Street, Room E5523, Baltimore, MD 21205 USA; 2 Health Services Academy, Chak Shahzad, Islamabad, Pakistan; 3 Human Development Research Foundation, Rwalpindi, Pakistan; 4 Institute of Population Health, University of Liverpool, Liverpool, UK

**Keywords:** Psychology, Medical research

## Abstract

Antenatal anxiety is among the risk factors for adverse birth outcomes, which are common in Pakistan. Between 2019 and 2022, we conducted a randomized controlled trial to evaluate the effects of the *Happy Mother-Healthy Baby* program, designed to reduce anxiety during pregnancy through use of Cognitive Behavior Therapy, on birth outcomes with 796 women in Rwalpindi, Pakistan. We performed intent-to-treat analysis and per protocol analyses. Intention-to-treat analyses showed no difference in the odds of low birthweight (LBW) (Adj. OR = 0.82, 95% CI 0.55–1.28 p = 0.37), preterm birth (PTB) (Adj. OR = 1.20 95% CI 0.83–1.71, p = 0.33) or small-for-gestational age (SGA) birth, (Adj. OR = 0.76, 95% CI 0.56–1.09, p = 0.16). Among completers who received ≥ 5 intervention sessions, the odds of LBW and SGA were 39% and 32% lower (Adj. OR = 0.61, 95% CI 0.43–0.87, p < 0.01; Adj. OR = 0.68, 95% CI 0.53–0.89, p < 0.01). The significant LBW and SGA results among the intervention completers suggest that the program may be effective when a sufficient dose is received. However, confirmation of these findings is needed due to the fact that randomization is not maintained in completer analyses.

**Clinical Trial Registration:** ClinicalTrials.gov Identifier: NCT03880032, 19/03/2019.

## Introduction

Adverse birth outcomes occur at high rates in low- and middle-income countries (LMICs)^1–3^. Of the 20.5 million children born with low birthweight (LBW) in 2015, 48% occurred in southern Asia^4^. Specifically in Pakistan, recent estimates showed that 47% of infants were born small-for-gestational age (SGA)^3^, 16% had pre-term birth (PTB)^3^, and 22% were born with LBW^5^. Compared to those born at term with normal birthweight, preterm and LBW infants are at higher risk for neonatal and post­neonatal morbidity and mortality^6–8^, poor cognitive functioning^7,9^, and chronic health and social disabilities^10–13^.

Antenatal anxiety is among the risk factors for poor fetal growth and PTB^14^. According to a study by Ciesielski and colleagues conducted in the United States, mothers who experienced antenatal anxiety had three times the odds of delivering an infant with poor fetal growth^15^. A systematic review and meta-analysis by Ding et al. found that across 12 studies conducted predominately in the United States and Europe, antenatal anxiety was significantly associated with a 50% higher risk of PTB and across six studies also conducted primarily in the United States and Europe, antenatal anxiety increased the risk of LBW by 76%^14^. In another systematic review, Staneva et al. found that even subclinical symptoms of anxiety were predictive of PTB and that birth outcomes were affected even by sub-threshold levels of anxiety^16^. Results of a study examining the timing of exposure suggests that anxiety even as early as the second trimester of pregnancy could increase the risk of SGA at birth^17,18^.

Given that interventions for postpartum common mental health disorders during pregnancy often start later in pregnancy or in the postpartum period^19,20^, it is possible that these interventions may start too late to avert poor growth in utero. For example, an evidence-based program to reduce postpartum depression in Pakistan, the *Thinking Healthy Program* (THP), began in the third trimester^21^. Although birth outcomes were not measured in the THP trial, the intervention did not improve child growth in the first year of life, a finding possibly attributable to the late initiation of the intervention^21^.

Because poor birth outcomes are highly prevalent in Pakistan^3,5^, this setting provides a unique opportunity to evaluate an intervention that could improve them. Given that antenatal anxiety and psychosocial stressors are associated with LBW and PTB, we hypothesized that improving maternal mental health during pregnancy could have a beneficial effect on birth outcomes. Therefore, we evaluated whether *Happy Mother, Healthy Baby* (HMHB), a Cognitive Behavior Therapy (CBT) based psychosocial intervention designed to prevent anxiety symptoms in pregnancy, could improve birth outcomes among Pakistani women experiencing at least mild symptoms of anxiety. Specifically, we hypothesized that randomization to the intervention would reduce the likelihood of LBW, PTB, and SGA at the time of delivery compared to enhanced usual care.

## Methods

### Participants

This study was a phase three, two-arm, single-blind, individual randomized clinical trial conducted between April 2019 and October 2022 in Rawalpindi, Pakistan (ClinicalTrials.gov identifier: NCT03880032 19/03/2019). Rawalpindi is a densely populated urban center in the Punjab province in northern Pakistan. 1,200 women were recruited from the Obstetrics and Gynecology outpatient department of Holy Family Hospital (HFH), a large public tertiary care facility affiliated with Rawalpindi Medical University. The women were approached during routine antenatal visits for initial screening and consent using the following inclusion criteria: age ≥ 18 years, gestational age ≤ 22 weeks, residence ≤ 20 km from HFH, intent to remain in the area through delivery, and ability to speak Urdu. After initial screening, a secondary eligibility screening took place involving the administration of the Hospital Anxiety and Depression Scale (HADS) and the Structured Clinical Interview for DSM-IV (SCID-4) was used to rule out Major Depressive Disorder conducted by non-specialist master’s level staff trained in psychology but with no prior clinical experience. These non-specialist providers (NSPs) were trained by a SCID-4-trained psychiatrist and achieved good inter-rater reliability. The HADS has been previously adapted and validated in Urdu^23,24^. Inclusion criteria from this secondary screening consisted of: evidence of at least mild anxiety (a score ≥ eight on the HADS anxiety subscale) and the absence of clinical depression or other serious medical conditions. Potential participants were excluded based on: a diagnosis of a major depressive episode, suicidal ideation, self-report of present or past psychiatric disorders (e.g., bipolar disorder, schizophrenia) or psychiatric care (e.g., anxiolytic medications, psychotropic drugs), or major physical health problems such as hepatitis and tuberculosis.

We excluded participants with these conditions as well as with clinical depression for two main reasons. First, although in real-world implementation studies (for example employing effectiveness and implementation-evaluation hybrid designs) less restrictive exclusion criteria would have been desirable, this was an efficacy trial of a new intervention targeting women with anxiety. Therefore, a key objective was to evaluate the impact of an intervention specifically for reducing anxiety in pregnancy in order to prevent postnatal depression. Second, given that birth outcomes were among our main outcomes of interest, and that prenatal depression is one of the strongest predictors of postpartum depression and is an independent risk factor for intrauterine growth restriction, including women with this condition would make the specific effects of reducing symptoms of anxiety on these birth outcomes unclear.

Ethical approval for this research was obtained from the Institutional Review Boards of Rawalpindi Medical University, Human Development Research Foundation, the Johns Hopkins Bloomberg School of Public Health, and a US National Institute of Mental Health (NIMH) appointed Data Monitoring and Safety Board. Study procedures and activities were performed in accordance with the relevant guidelines and regulations from these institutions. All participants were given verbal and written information about the study prior to recruitment, and provided written informed consent prior to screening and data collection.

### Procedures

Participants were randomly assigned to one of two treatment arms, intervention or enhanced care. Prior to participant enrollment, the trial statistician constructed randomly permuted blocks of size 4, 8, 12, and 16 with arm assignment using a pseudo random-number generator. The assignment list was printed in order, with each step of the sequence separately stored in opaque envelopes and numbered sequentially with a seven-digit code. Once an eligible woman provided consent for study participation, the next available envelope was pulled and opened, and assignment to intervention or control group was recorded. This procedure continued until desired sample size (600 in each arm) was reached. The study over-enrolled in each arm to account for an expected 30% attrition. The target for complete data was 840 (420 per arm). A descriptive table for all enrolled participants is detailed in Surkan et al.^25^.

If a participant was randomly assigned to the intervention arm, she was scheduled to receive six core sessions and up to six booster sessions of HMHB in addition to regular antenatal care^26^. The first five sessions were delivered on a weekly basis immediately after enrollment and the sixth in the third trimester. Booster sessions were placed between the fifth and sixth session (as deemed necessary by the therapist and based on whether time was available before delivery). Based on extensive qualitative formative research prior to the trial^26^, HMHB was designed for pregnant women experiencing anxiety during early to mid-pregnancy. It employed the core principles and strategies of a World Health Organization-endorsed evidence- based intervention for perinatal depression called the *Thinking Healthy Program* (THP)^27^. HMHB was aimed at improving recipients’ personal wellbeing, bonding with the baby and social support (e.g., family), through developing empathetic relationships, challenging and replacing unhelpful thoughts, behavioral activation, enhancing problem solving skills and relaxation exercises^26^. Intervention materials were customized with culturally relevant illustrations and scenarios to assist with guided discovery, behavior activation, stress management, and to convey key health messages^26^. For further detail on the development and adaptation of HMHB, please refer to Atif et al.^26^. Sessions were delivered by NSPs with four years of university level education (a two-year bachelor’s and a two-year master’s degree in psychology, but no clinical training). Prior to delivering the intervention, NSPs participated in at least 42 h of training on anxiety and its consequences, counseling skills, and the delivery of HMHB. All NSPs were supervised by a PhD-level specialist trainer and participated in weekly supervision sessions. Over the course of the study, 252 (12%) of sessions were randomly selected and assessed for quality using the Enhancing Assessment of Common Therapeutic factors (ENACT) system^28^.

The intervention was delivered to study participants in a series of one-on-one sessions at the hospital and complemented by take-home exercises. Sessions one, three and six involved significant family members like the husband or mother-in-law, when possible (if accompanying the participant to the hospital). The first session introduced the HMHB program and focused on rapport-building between the mother and NSP. The second session aimed to discuss the connection between thoughts and actions and how the mother can use this connection to manage stress and improve personal well-being. The third session focused on identifying sources of social support and how the mother would be supported during her pregnancy and after childbirth. The fourth session centered on the relationship between the mother and her unborn baby and provided an opportunity for the mother to identify and discuss any concerns about childbirth. The fifth session involved reviewing concepts from the previous four sessions and developing a plan for the mother to maintain practices learned over the course of the intervention. The last session focused on discussing potential postnatal challenges and how skills learned throughout the program could be used to manage them. Breathing exercises for relaxation were also taught and practiced throughout the intervention sessions. Due to the COVID-19 pandemic occurring in the middle of data collection for this study, therapy sessions and assessments were moved to the phone for some participants. Both treatment and control group participants received reimbursement for transportation and ultrasounds, and facilitation in receiving care (e.g., expediated check-ups). In addition to these enhancements in care, the control arm received antenatal checkup visits as usual.

### Outcomes

The primary outcomes for the intervention overall^29^ were common mental disorders of anxiety and depression. The analyses conducted for this article address the secondary outcomes of LBW (yes/no), PTB (yes/no), and SGA (yes/no) taken from hospital medical records. Anthropometric measurements (e.g., newborn weight and length) were assessed immediately after childbirth by nurses in the hospital. Birthweight was measured using the LAICA PS3004 scale with precision to 0.1 kg. Each of the outcomes was assessed using World Health Organization guidelines^30^; LBW was defined as < 2.5 kg (kg), PTB was defined as < 37 weeks’ gestation, and SGA was defined as weight at birth < 10th percentile for gestational age. Stillbirth was defined as fetal death after 28 weeks’ gestation or during delivery and neonatal death was defined as death of the infant within the first 28 days of life. Information on demographic variables assessed at baseline included: age, education, family structure, migration status, whether the current pregnancy was the participant’s first pregnancy, and history of miscarriage or stillbirth. Additional details on study protocol can be found in Surkan et al.^29^.

### Statistical analyses

Among pregnancies with known birth outcomes, we examined the differences at baseline between randomized arms, to verify the randomization generated comparable groups. We used standard statistical comparisons, including the Chi-squared and the Student’s t-test, to determine the statistical significance of any differences between arm. Unless otherwise noted, all analysis follows the intent-to-treat (ITT) principle, comparing pregnancies and births in the groups to which they were randomized. While we considered LBW and PTB also as birth outcomes of interest, we chose one outcome to base our sample size on, i.e. SGA. An a priori power analysis was conducted to determine the minimum sample size required to test the reduction of SGA incidence of 20.2%, under the assumption of 80% power, a significance criterion of alpha = 0.05, and SGA prevalence in the population of 47%^3^. Under these assumptions, the sample size needed was N = 420 women per arm. It should be noted that the analyses may be underpowered due to the fact that prevalence of SGA was lower than our estimates from the prior literature and because more participants than anticipated were lost to follow-up (likely due to the interference of the COVID-19 pandemic during data collection).

We compared birthweight, gestational age, stillbirth, miscarriage, and neonatal mortality rates between arms using non-parametric tests for statistical inference, with Fisher’s Exact test for dichotomous outcomes and Wilcoxon Rank Sum for continuous outcomes birthweight and gestational age due to anticipated non-standard distributions and the potential for low counts. For birthweight, we compared weight at birth in kilograms using linear regression, as well as whether livebirths were less than 2.5 kg using logistic regression. We also estimated differences between arms, where possible, also adjusting for maternal age, income, education, and gravidity, and child sex. We also examined the intervention effect among those receiving five or six intervention sessions compared to those in the control arm, adjusting for age, education, income, gravidity, women’s empowerment, relationship quality (MRQ) and social support (MSPSS) using a propensity score^31^. In this analysis, we defined receipt of at least five of the six core HMHB sessions as the complete dose due to the fact that some women with preterm, SGA and low birthweight births would not have the opportunity to have the sixth visit since it was delivered in the third trimester.

### Ethics approval

Ethics approval was obtained from the Johns Hopkins Bloomberg School of Health Institutional Review Board (Baltimore, USA), the Human Development Research Foundation Ethics Committee (Islamabad, Pakistan), the Rawalpindi Medical University (RMU) Institutional Research Forum (Rawalpindi, Pakistan) and the U.S. National Institute of Mental Health-appointed Global Mental Health Data Safety and Monitoring Board.

## Results

**Figure 1:**
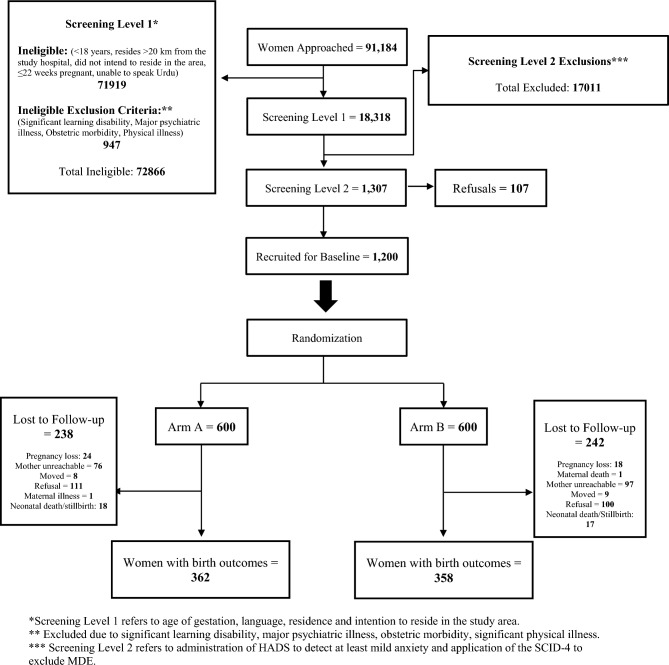
Consort chart.

Out of over ninety-one thousand women screened, 1307 women met the inclusion criteria, including having mild, moderate, or high anxiety symptoms and not meeting the diagnostic criteria for clinical depression on the HADS. Of these 1200 (92%) agreed to participate and were enrolled in the trial. Among the 1200 pregnant women who were enrolled and randomized to receive either the HMHB intervention or enhanced care, 404 (34%) were lost to follow-up, mostly because they were unreachable or because they refused participation (Fig. 1). The remaining 796 (66%) of enrolled pregnant women were followed until birth, including miscarriages, stillbirths, and livebirths. There were 720 (60%) live births and 76 (6%) miscarriages or stillbirths.

Pregnant women with known birth outcomes were similar at enrollment across all examined features between arms. These included having a male child (49% versus 49%, p = 0.88), maternal age, whether it was the participant’s first pregnancy, and whether the participant had a history of stillbirth or miscarriage. Education and self-reported monthly income were also similar between arms. Participant baseline anxiety and depression symptoms were similar between arms. A detailed description of participants by arm is shown in Table 1.

**Table 1:** Description of 720 pregnant women with birth outcomes in the HMHB trial at enrollment.

	Overall (N = 720)	Intervention arm (N = 362)	Control arm (N = 358)	p*
	Mean (SD)	Mean (SD)	Mean (SD)	
Age (years)	25.2 (4.6)	25.1 (4.7)	25.3 (4.5)	0.52
Gestational age (weeks)	15.8 (4.5)	15.7 (4.5)	15.9 (4.5)	0.71
	N (%)	N (%)	N (%)	
Child sex (male)	352 (49%)	178 (49%)	174 (49%)	0.94
Maternal age ≤ 25 years	337 (47%)	172 (48%)	165 (46%)	0.76
First pregnancy (yes)	207 (29%)	98 (27%)	109 (30%)	0.36
Residing with at least one child	411 (57%)	204 (56%)	207 (58%)	0.75
History of stillbirth or miscarriage (yes)	303 (42%)	160 (44%)	143 (40%)	0.28
Migrant status (yes)	529 (73%)	257 (71%)	272 (76%)	0.15
Education level				
≤ Primary school	177 (25%)	92 (25%)	85 (24%)	0.87
Middle school—matriculation	336 (47%)	167 (46%)	169 (47%)	
≥ Intermediate	207 (29%)	103 (28%)	104 (29%)	
Family structure				
Nuclear	231 (32%)	119 (33%)	112 (31%)	0.86
Joint (parents)	245 (34%)	120 (33%)	125 (35%)	
Extended (parents and siblings)	244 (34%)	123 (34%)	121 (34%)	
Monthly income (PKR)				
Low (< 20,000)	318 (44%)	156 (43%)	162 (45%)	0.67
Middle (20,000–35,000)	285 (40%)	143 (40%)	142 (40%)	
High (> 35,000)	117 (16%)	63 (17%)	54 (15%)	
Anxiety at enrollment (HADS)	11.2 (1.9)	11.2 (2.0)	11.2 (1.9)	0.93
Depression at enrollment (HADS)	6.7 (2.8)	6.9 (2.9)	6.6 (2.6)	0.21
Stress at enrollment (PSS-10)	19.9 (2.5)	20.0 (2.7)	19.8 (2.4)	0.52
Significant other support (MSPSS)	3.3 (0.9)	3.3 (0.9)	3.4 (0.8)	0.32
Women’s empowerment	366 (51%)	174 (48%)	192 (54%)	0.16
Marital relationship questionnaire (MRQ)				
Emotional	2.7 (1.0)	2.7 (1.0)	2.7 (0.9)	0.45
Instrumental	2.5 (1.0)	2.5 (1.0)	2.5 (1.0)	0.80
Conflict	1.0 (1.1)	1.1 (1.1)	1.0 (1.1)	0.08

*PKR* Pakistani Rupees.

*Significance by Student’s t test for continuous factors, Chi-square test for categorical factors.

We examined birthweight among livebirths, both continuously in kilograms, and also as a dichotomous measure, being < 2.5 kg. Birthweight in kilograms was similar between arms (2.9 versus 2.8, p = 0.48, Table 2), with an estimated difference of 0.02 (95% confidence interval (CI) − 0.06 to 0.09, p = 0.66) using linear regression, after adjustment for maternal age, education, gravidity, and income, and child sex. The likelihood of having birthweight < 2.5 kg was also similar between arms, with 12.7% having LBW in the intervention arm, and 14.8% in the control arm (p = 0.45), having an odds ratio (OR) of 0.82 (95% CI 0.55–1.28, p = 0.37, adjusted for maternal age, education, income, gravidity, and child sex). Details including unadjusted odds ratios are shown in Table 3.

**Table 2:** Differences in birth outcomes between intervention arms among 720 women in the HMHB trial.

	N (overall)	Intervention arm Median (IQR) or N (%)	Control arm Median (IQR) or N (%)	p* (difference)
Child weight at birth (kg)	720	3.0 (2.5, 3.1)	3.0 (2.5, 3.1)	0.48
Child birthweight < 2.5 kg	720	46 (12.7%)	53 (14.8%)	0.45
Gestational age at birth (weeks)	720	39.0 (36.0, 40.0)	38.0 (37.0, 39.0)	0.98
Gestational age at birth < 37 weeks	720	79 (21.8%)	68 (19.0%)	0.36
Small-for-gestational-age at birth	720	89 (24.6%)	105 (29.3%)	0.15

*Significance determined by and Fisher exact test for all features except for birth age and birth weight, which used the Wilcoxon rank sum to compare the median between arms.

**Table 3:** Estimated intervention effects (intervention arm relative to control arm) among 720 women with birth outcomes in the HMHB trial.

	Unadjusted			Adjusted*		
	Est	95% CI	p	Est	95% CI	p
Child weight at birth (kg)	0.01	(− 0.06, 0.09)	0.71	0.02	(− 0.06, 0.09)	0.66
Child birth weight < 2.5 kg^+^	0.84	(0.55, 1.28)	0.41	0.82	(0.55, 1.28)	0.37
Gestational age at birth (weeks)	− 0.07	(− 0.36, 0.23)	0.65	− 0.07	(− 0.36, 0.23)	0.62
Gestational age at birth < 37 weeks^+^	1.19	(0.83, 1.71)	0.35	1.20	(0.83, 1.71)	0.33
Small-for-gestational-age at birth^+^	0.79	(0.56, 1.09)	0.15	0.76	(0.56, 1.09)	0.12

*CI* confidence interval, *NE* not estimable.

^+^Logistic regression. Significance determined by logistic regression.

*Adjusted for maternal age, education, first pregnancy, and income, and child sex, using linear or logistic regression.

Gestational ages were similar between arms among 720 livebirths (38.0 weeks in the intervention arm and 38.2 weeks in the control arm, p = 0.98). Gestational ages were also similar between arms when examining whether there was prematurity (gestational age < 37 weeks, 21.8% versus 19.0%, p = 0.36). A description of gestational age by arm is shown in Table 2. These estimates of differences between arms and using linear regression were adjusted for maternal age, income, education, and gravidity, and child sex (Table 3). The likelihood of being SGA was similar between arms, with 24.6% being SGA in the intervention arm and 29.3% in the control arm, with an OR of 0.76 (95% CI 0.56–1.09, p = 0.12), adjusted for maternal age, education, income, gravidity, and child sex. There were no changes in significance when using linear versus logistic regression models.

**Figure 2:**
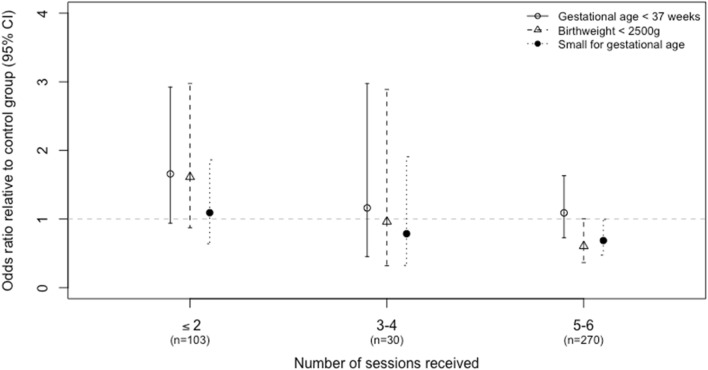
Odds ratio for small for gestational age by the number of intervention sessions received, for the intervention arm relative to the control arm. The number of births in the intervention group is shown for each number of doses, compared to all births in the control arm (n = 358). Odds ratios are adjusted for maternal age, income, education, and first pregnancy, and child sex using logistic regression.

In addition to our primary analysis, which was completed following the principle of intent to treat, we conducted additional analyses excluding those in the intervention arm with fewer than five intervention sessions. We adjusted for potential differences in this group and the control arm using a propensity score analysis, which included maternal age, education, income, gravidity, women’s empowerment, and significant other support (MSPSS). These results are depicted in Fig. 2. For those receiving at least five intervention sessions, the odds ratio for having LBW (less than 2.5 kg) was marginally lower and statistically significant compared to the control arm (adjusted OR = 0.61, 95% CI 0.43–0.87, p < 0.01), as was SGA (adjusted OR = 0.68, 95% CI 0.53–0.89, p < 0.01). These results are shown in Table 4 by dosage of number of sessions received, each compared to a similar group of control arm participants determined through the propensity score analysis.

**Table 4:** Estimated intervention effects (intervention arm relative to control arm) among 358 women in the control arm, and 257 women in the intervention arm receiving five or six intervention sessions in the HMHB trial.

	Unadjusted			Adjusted*		
	Est	95% CI	p	Est	95% CI	p
Child weight at birth (kg)	0.06	(− 0.02, 0.15)	0.14	0.07	(− 0.02, 0.15)	0.10
Child birthweight < 2.5 kg^+^	0.62	(0.37, 1.03)	0.06	0.61	(0.43, 0.87)	< 0.01
Gestational age at birth (weeks)	0.07	(− 0.24, 0.39)	0.64	0.04	(− 0.24, 0.39)	0.78
Gestational age at birth < 37^+^ weeks	1.08	(0.72, 1.62)	0.70	1.08	(0.82, 1.44)	0.58
Small for gestational age^+^	0.70	(0.48, 1.02)	0.06	0.68	(0.53, 0.89)	< 0.01

*CI* confidence interval, *NE* not estimable.

^+^Logistic regression. Significance determined by logistic regression.

*Adjusted for maternal age, education, income, first pregnancy, women’s empowerment, and significant other support (MSPSS), with a propensity score.

Miscarriage, stillbirth, and neonatal death were also compared between arms. Rates overall for these among livebirths in the trial were low, as expected, at 5.2%, 2.1%, and 4.4%, respectively. Estimated differences between arms were not statistically different from zero (Data not shown).

## Discussion

In the intention to treat analysis, participation in the intervention did not produce any statistically significant change in LBW, PTB, or SGA outcomes. However, women who completed at least five of the sessions had 39% lower odds of low birthweight (< 2.5 kg), and 32% lower odds of SGA in the intervention group, both of which attained statistical significance. These findings suggest that the number of intervention sessions, i.e., sufficient intervention dose, may be important in reducing LBW and SGA in women with anxiety.

This study contributes to the literature in important ways. First, despite the high burden of poor maternal mental health and its association with child outcomes in Asia, minimal research on interventions to address antenatal anxiety and birth outcomes has been conducted. To date, we know only of anxiety interventions in higher resource contexts such as the United States and Europe that have studied birth outcomes^32–34^, suggesting that more research in lower resource contexts, such as Pakistan, is needed.

In line with our findings of no effect on birth outcomes in the ITT analysis, a meta-analysis of randomized control trials conducted by Brouwer et al. showed that when pooled, interventions targeting either antenatal anxiety, depression, or depression and anxiety had no statistically significant effect on improving birthweight or gestational age^35^. In this meta-analysis, the three interventions that focused solely on antenatal anxiety found mixed results. Bastani et al. tested an applied relaxation training delivered once a week over seven weeks in a group setting in Iran. This study found a statistically significant increase in mean birthweight between the intervention and control group, though no difference in mean gestational age^36^. Using a similar relaxation intervention delivered at the individual level in the United States, Chambers found no difference in mean birthweight, gestational age, or APGAR scores in a sample of women^37^. Similar to Chambers, Cappon found no difference in mean birthweight after testing an individually delivered intervention where pregnant women at various stages of pregnancy listened to music to relax for 15–20 min per session for a minimum of 20 sessions throughout the pregnancy^38^. Like these studies, our intervention also included a relaxation component through breathing exercises, however our main intervention strategy was based on cognitive behavior techniques.

As antenatal depression often coexists with anxiety, CBT intervention studies targeting antenatal depression (rather than anxiety) have shown mixed results on birth outcomes. Netsi et al. conducted a randomized control trial among women with depression in the UK and found no difference in infant birthweights between the treatment group receiving 12 in-home CBT sessions and the treatment as usual group^39^. Similarly, Verbeek found no effects on birthweight or gestational age in their randomized control trial of CBT that compared to treatment as usual among women in the Netherlands^40^. Contrary to the previous two studies, Karamoozian and Askarizadeh found statistically significant improvement in newborn APGAR scores among a group of Iranian women who received a cognitive-behavioral stress management intervention prior to giving birth compared to those in the control group^41^. Given the limited number of intervention studies targeting either antenatal anxiety or antenatal depression, especially in LMICs, more research is needed to understand and address complex mechanisms responsible for birth outcomes.

In addition to psychosocial interventions, nutrition, deworming, maternal education, and water sanitation and hygiene (WASH) interventions have been evaluated for their effects on LBW and PTB^42^. Park et al. conducted a systematic review and meta-analysis of interventions to improve birth outcomes in LMICs. Regarding both PTB and LBW, only interventions focused on intake of 1500 kcal of local food per day were found to show a statistically significant improvement of PTB outcomes. Other types of interventions (e.g., WASH, education, and deworming) demonstrated trends in reducing PTB and LBW, but did not show statistically significant differences^42^. Synthesizing others’ results with the findings of our study, the improvement of birth outcomes is a complex public health challenge and more research is needed. It may also be the case that interventions could benefit from combining multiple interventions across different domains, e.g., mental health and nutrition, to provide a more holistic intervention package.

This study is not without limitations. First, the descriptive statistics showed 151 cases (21%) of the sample with a birthweight of exactly 2.5 kg, suggesting that the precision of the scale (to 0.1 kg) or rounding may have precluded more accurate birthweight measurements. This may have diluted the magnitude of the effects we saw in the ITT analysis. Second, we had a large number of enrolled participants who were lost to follow-up (N = 404 or 34%). This may partially be explained by the COVID-19 pandemic, which impacted women’s perceptions of health care as they had heightened mistrust of health care professionals and increased fear of infection while accessing care in a government hospital^43^. However, we lacked data to quantitatively assess the influence of the COVID-19 pandemic on dropout. Our process evaluation indicated that low family engagement and stigma of mental health also posed barriers to retention in the study^44^. Given the loss to follow-up, we may have been underpowered to detect an intervention effect in the ITT analysis. Another contributing factor to our high loss to follow-up may have been that we did not use strategies to limit post-randomization withdrawals such as delaying randomization until participants returned for the next hospital visit. Lastly, we conducted an analysis among those in the intervention arm completing five or six sessions in comparison to the control group with a robust approach to adjust for potential confounding. However, confounding from unmeasured factors may still exist for which we could not adjust, suggesting the possibility of alternative explanations and that further study is needed to confirm our findings. In other words, in completer analyses the benefits of the randomization no longer hold since the study arms are no longer randomized^45^. Other disadvantages are that completer analyses only provide information about the performance of the intervention in an ideal scenario^45^, for example, when participants received the full treatment.

Although our results were mixed and our study had important limitations, more research should be done to confirm these results. To ensure more accuracy of birthweights in medical records, higher precision scales should be used when possible, research teams should train hospital personnel regarding rounding errors or have a quality check or researcher present to ensure accurate recording. Nonetheless, our results suggest that receipt of the full dose of treatment of an anxiety focused intervention (at least five sessions) may reduce the risk of LBW and SGA among anxious women. Upon confirming the efficacy of the full dose of HMHB on birth outcomes, this intervention could be then further evaluated through an effectiveness trial, and ultimately integrated into routine practice. Given the paucity of research in South Asia examining the effects of mental health interventions on birth outcomes, this study provides an important preliminary contribution to the literature (Supplementary Table S1).

### Supplementary Information

Supplementary Table 1.

## Data Availability

The data associated with this study has been uploaded to the National Institutes of Health, National Institute of Mental Health’s Data Archive (https://nda.nih.gov/edit_collection.html?id=3751) for public use.
