# Supplementary material for: Results of a cognitive behavior therapy-based intervention for antenatal anxiety on birth outcomes in Pakistan: a randomized control trial
Source: Sci Rep. 2024 Jun 14;14:13806. doi: 10.1038/s41598-024-64119-z (PMC11178914; doi:10.1038/s41598-024-64119-z)
Supplement: Supplementary file 1 — Supplementary Table 1. [file 41598_2024_64119_MOESM1_ESM.docx]

**Supplemental Table 1. Description of among 358 women in the control arm, and 257 women in the intervention arm receiving five or six intervention sessions in the HMHB trial.**

|  | **Overall**  **(N=615)** | **Intervention**  **Arm**  **(N=257)** | **Control**  **Arm**  **(N=358)** | **p*** |
| --- | --- | --- | --- | --- |
|  | Mean (SD) | Mean (SD) | Mean (SD) |  |
| Age (years) | 25.4 (4.7) | 25.4 (4.8) | 25.3 (4.5) | 0.91 |
| Gestational Age (weeks) | 15.8 (4.5) | 15.6 (4.4) | 15.9 (4.5) | 0.53 |
|  |  |  |  |  |
|  | N (%) | N (%) | N (%) |  |
| Child sex (male) | 298 (48%) | 124 (48%) | 174 (49%) | 1.00 |
| Maternal age ≤25 years | 286 (47%) | 121 (47%) | 165 (46%) | 0.87 |
| First pregnancy (Yes) | 173 (28%) | 64 (25%) | 109 (30%) | 0.16 |
| Residing with at least one child | 360 (59%) | 153 (60%) | 207 (58%) | 0.73 |
| History of stillbirth or miscarriage (Yes) | 256 (42%) | 113 (44%) | 143 (40%) | 0.36 |
|  |  |  |  |  |
| Migrant status (Yes) | 458 (74%) | 186 (72%) | 272 (76%) | 0.36 |
| Education level |  |  |  | 0.62 |
| ≤ Primary school | 154 (25%) | 69 (27%) | 85 (24%) |  |
| Middle school – matriculation | 289 (47%) | 120 (47%) | 169 (47%) |  |
| ≥ Intermediate | 172 (28%) | 68 (26%) | 104 (29%) |  |
| Family structure |  |  |  | 0.39 |
| Nuclear | 205 (33%) | 93 (36%) | 112 (31%) |  |
| Joint (parents) | 206 (33%) | 85 (33%) | 121 (34%) |  |
| Extended (parents and siblings) | 204 (33%) | 79 (31%) | 125 (35%) |  |
| Monthly income (PKR) |  |  |  | 0.95 |
| Low (<20,000) | 276 (45%) | 114 (44%) | 162 (45%) |  |
| Middle (20,000-35,000) | 244 (40%) | 102 (40%) | 142 (40%) |  |
| High (>35,000) | 95 (15%) | 41 (16%) | 54 (15%) |  |
|  |  |  |  |  |
| Anxiety at enrollment (HADS) | 11.1 (1.9) | 11.1 (2.0) | 11.2 (1.9) | 0.95 |
| Depression at enrollment (HADS) | 6.7 (2.7) | 6.9 (2.9) | 6.6 (2.6) | 0.17 |
| Stress at enrollment (PSS-10) | 19.9 (2.5) | 20.0 (2.6) | 19.8 (2.4) | 0.52 |
|  |  |  |  |  |
| Significant other support (MSPSS) | 3.3 (0.9) | 3.3 (1.0) | 3.4 (0.8) | 0.18 |
| Women’s empowerment | 316 (51%) | 124 (48%) | 192 (54%) | 0.22 |
| MRQ (emotional) | 2.7 (1.0) | 2.7 (1.0) | 2.7 (0.9) | 0.73 |
| MRQ (instrumental) | 2.5 (1.0) | 2.5 (1.0) | 2.5 (1.0) | 0.65 |
| MRQ (conflict) | 1.0 (1.1) | 1.1 (1.1) | 1.0 (1.1) | 0.15 |

* Significance by Student’s t test for continuous factors, Chi-square test for categorical factors.

Note: PKR refers to Pakistani Rupees
